# The first complete mitogenome of skin beetles *Omorgus chinensis* (Coleoptera: Trogidae) with the phylogenetic implications

**DOI:** 10.1080/23802359.2021.2008823

**Published:** 2021-12-19

**Authors:** Hao Yu, Yong-jing Chen, Ying Ying, Jiao-Jiao Yuan

**Affiliations:** a Department of Entomology, School of Resources and Environmental Engineering, Henan Institute of Science and Technology, Xinxiang, Henan, P. R. China; b Department of Ecology, School of Resources and Environmental Engineering, Anhui University, Hefei, Anhui, P. R. China

**Keywords:** *Omorgus chinensis*, Trogidae, mitogenome, phylogenomic analysis

## Abstract

The first complete mitogenome of Trogidae, *Omorgus chinensis* (Coleoptera: Trogidae) is sequenced using the next generation sequencing. The genomic structure is a circular molecule with 18682 bp in length, comprising 13 protein-coding genes, 22 transfer RNA genes (tRNAs), 2 ribosomal RNAs (rRNAs) and a control region. The nucleotide composition is A (39.44%), C (13.82%), T (36.78%), and G (9.96%) with an AT content of 76.22%. The phylogenetic analysis of 18 insects in Scarabaeoidae show that *Omorgus chinensis* shares a close ancestry with Lucanidae and Geotrupidae.

## Introduction

*Omorgus chinensis* (Coleoptera: Trogidae) is an important necrophagous insect with an oval shape and tan color. Like most members in Trogidae, this species has a marked engraved and dimpled area on the anterior thoracic plate and a strong arch on the back but a flattened ventral surface (Ren 2003). In recent years, with the development of molecular biology, especially the advancement of next-generation sequencing technology, mitochondrial genome (mitogenome) have been widely used as a typical marker for the phylogenetic study of coleopteran insects (Cameron 2014; Li et al. 2015; Timmermans et al. 2015). Fagua et al. 2018; However, there are only a few molecule phylogenetic researches in Trogidae (Strümpher et al. 2014), and no complete mitogenome data has yet been sequenced.

In this study, we sequenced the complete mitogenome of *Omorgus chinensis* for the first time using the next-generation methods and reconstructed the phylogenetic relationships within other species in Scarabaeoidae, which provides reliable data for subsequent further phylogenetic studies.

## Materials and methods

### Sample collection and DNA extraction

The specimens of *O. chinensis* were collected by Yongjing Chen and Shiju Zhou (2289788957@qq.com) from Yaoluoping National Nature Reserve, Anhui province, P. R. China, in July 2017. For adult specimens, the muscle tissues were placed in 100% ethanol. Total genomic DNA was extracted from the muscle of a single *O. chinensis* using the Qiagen DNAeasy Kit. The specimen deposited in the Museum of Anhui university and the sequence was submitted to GenBank with the accession number MK937809.

### Polymerase chain reaction amplification, sequencing and assembly

Complete mitogenome was assembled by overlapping high-throughput fragment sequences, the amplification of the three primers were used as markers for tandem high-throughput sequencing fragment sequences. The primers used for amplification listed in Table 1. PCR amplification reactions were carried out in 25 μL volumes containing 10 μM of each primer (forward and reverse) 1 μL, 2 μL template DNA, 12.5 μL 2 × EasyTaq SuperMix (+dye), and 8.5 μL sterile double-distilled water to make up a final volume of 25 μL. The polymerase chain reaction amplifications were performed under the following conditions: an initial denaturation at 94 °C for 2 min, followed by 35–37 cycles of denaturation at 94 °C for 40 seconds, annealing at 52–58 °C for 50 seconds, and elongation at 70 °C for 1 min, and then a final extension step at 72 °C for 7 min. The temperature of annealing was determined by the length of fragments. An Illumina TruSeq library was prepared with an average insert size of 450 bp and was sequenced using the Illumina Hiseq2500 platform with 250 bp paired-end reads. Raw reads were trimmed of adapters using Trimmomatic. Low quality and short reads were removed with Prinseq. High quality reads were used in denovo assembly using IDBA-UD with minimum and maximum k values of 80 and 240 bp, respectively. To investigate the accuracy of the assembly, clean reads were mapped onto the obtained mt contig using Geneious Prime 2019.1.1 (http://www.geneious.com/), allowing for mismatches of up to 2%, maximum gap size of 3 bp and minimum overlap of 100 bp. Finally, we obtained the mitogenome of *O. chinensis* with the average sequencing depth of 242×.

**Table 1.** Details on primers used in this study.

Gene	Prime name	Sequence(5’-3’)	Length	References
COI	COI-F1	CAACATTTATTTTGATTTTTTGG	23 bp	Simon et al. (1994)
	COI-R1	TCCAATGCACTAATCTGCCATATTA	25 bp	Simon et al. (1994)
Cytb	Cytb-F2	GAGGAGCAACTGTAATTACTAA	22 bp	Balke et al. (2004)
	Cytb-R2	AAAAGAAARTATCATTCAGGTTGAAT	26 bp	Balke et al. (2004)
rrnL	16S-F1	CCGGTTTGAACTCAGATCATG	21 bp	Hosoya et al. (2001)
	16S-R1	TAATTTATTGTACCTTGTGTATCAG	25 bp	Hosoya et al. (2001)

### Mitogenome annotation and analysis

Preliminary annotations made with the MITOS WebServer (Bernt et al. 2013) (http://mitos.bioinf.uni-leipzig.de/index.py). tRNA genes and their secondary structures were inferred using tRNAscan-SE 1.21 (Schattner et al. 2005) (http://lowelab.ucsc.edu/tRNAscan-SE/). Those not identified by tRNAscan-SE, in addition to 16S ribosomal RNA (rrnL, lrRNA), and 12S ribosomal RNA (rrnS, srRNA), were determined according to sequence similarity with related species. The protein-coding genes (PCGs) were determined by ORF Finder (http://www.ncbi.nlm.nih.gov/gorf/gorf.html) under the invertebrate mitochondrial genetic code. Nucleotide compositions, codon usage, and relative synonymous codon usage (RSCU) values of PCGs were calculated with MEGA-X (Kumar et al. 2018). PCGs were translated with DNAMAN v7.0.2.176 (Lynnon Biosoft, Vaudreuil-Dorion, Canada). Composition skew analysis was conducted according to formulas AT-skew = [A − T]/[A + T] and GC-skew = [G − C]/[G + C] (Perna and Kocher 1995).

### Phylogenetic analyses

A total of 18 mitochondrial genomes of Scarabaeoidae were used for phylogenetic analysis (Table 2). Each PCGs was aligned individually based on codon-based multiple alignments using the MEGA-X (Kumar et al. 2018). Models of nucleotide substitution were selected according to the Akaike Information Criterion (AIC) with jModelTest v2.1.4 (Posada 2008). Phylogenetic trees were generated from ML analysis using RAxML (Stamatakis 2014) and Bayesian inference (BI) with MrBayes v3.2.5 (Ronquist et al. 2012), both under the GTR + I + G model. Node support in the ML tree was estimated through bootstrap analysis with 1,000 replicates. The BI was conducted with two simultaneous Markov chain Monte Carlo runs of 2 × 10^9^ generations, sampled every 1,000 steps, with the first 25% discarded as burn-in. Phylogenetic trees were viewed and edited in Figtree v1.4.3 (Kim et al. 2021) (http://tree.bio.ed.ac.uk/software/figtree/).

**Table 2.** Taxa used in this study.

Family	Species	GenBank accession number
Aphodiidae	*Aphodius foetens*	KX087240
Cetoniidae	*Protaetia brevitarsis*	NC023453
Dynastidae	*Cyphonistes vallatus*	JX412731
Euchiridae	*Cheirotonus jansoni*	NC023246
Geotrupidae	*Anoplotrupes stercorosus*	JX412838
Hybosoridae	*Ceratocanthus sp.*	JX412772
Lucanidae	*Cyclommathus vitalisi*	MF037205
	*Dorcus curvidens hopei*	MF612067
	*Lucanus fortunei*	JX313688
	*Lucanus mazama*	NC013578
	*Prismognathus prossi*	MF614014
	*Prosopocoilus confucius*	KU552119
	*Prosopocoilus gracilis*	KP735805
Melolonthidae	*Rhopaea magnicornis*	NC013252
Rutelidae	*Popillia japonica*	MG971231
Scarabaeidae	*Onthophagus yukae*	KU739463
Sinodendridae	*Sinodendron yunnanense*	KP735804
Trogidae	Omorgus chinensis*	MK937809

*Mitochondrial genome sequenced in present study.

**Table 3.** Mitogenome organization of *Omorgus chinensis*.

Gene	Strand	Region	Length (bp)	Start codon	Stop codon	Anti-codon	Intergenic nucleotides (bp)
*trnI-Ile*	J	1–66	66	–	–	GAT	2
*trnQ-Gln*	N	69–137	69	–	–	TTG	5
*trnM-Met*	J	143–211	69	–	–	CAT	0
*nad2*	J	212–1225	1014	ATT	TAA	–	9
*trnW-Trp*	J	1235–1302	68	–	–	TCA	−8
*trnC-Cys*	N	1295–1357	63	–	–	GCA	0
*trnY-Tyr*	N	1358–1422	65	–	–	GTA	1
*COI*	J	1424–2959	1536	AAT	TAA	–	−5
*trnL(UUR)-Leu*	J	2955–3018	64	–	–	TAA	−2
*COII*	J	3017–3704	688	ATG	T	–	0
*trnK-Lys*	J	3705–3776	72	–	–	CTT	4
*trnD-Asp*	J	3781–3847	67	–	–	GTC	0
*atp8*	J	3848–4003	156	ATT	TAA	–	−4
*atp6*	J	4000–4671	672	ATA	TAA	–	−1
*COIII*	J	4671–5458	788	ATG	TA	–	−1
*trnG-Gly*	J	5458–5523	66	–	–	TCC	0
*nad3*	J	5524–5877	354	ATT	TAG	–	−2
*trnA-Ala*	J	5876–5942	67	–	–	TGC	0
*trnR-Arg*	J	5943–6009	67	–	–	TCG	−1
*trnN-Asn*	J	6009–6074	66	–	–	GTT	0
*trnS(AGN)-Ser*	J	6075–6140	66	–	–	TCT	1
*trnE-Glu*	J	6142–6204	63	–	–	TTC	−2
*trnF-Phe*	N	6203–6268	66	–	–	GAA	−1
*nad5*	N	6268–7988	1721	ATT	TA	–	0
*trnH-His*	N	7989–8053	65	–	–	GTG	−1
*nad4*	N	8053–9390	1338	ATG	TAA	–	−7
*nad4L*	N	9384–9674	291	ATG	TAA	–	2
*trnT-Thr*	J	9677–9739	63	–	–	TGT	0
*trnP-Pro*	N	9740–9804	65	–	–	TGG	1
*nad6*	J	9806–10309	504	ATC	TAA	–	0
*Cytb*	J	10310–11458	1149	ATA	TAG	–	−2
*trnS(UCN)-Ser*	J	11457–11522	66	–	–	TGA	18
*nad1*	N	11541–12491	951	ATT	TAG	–	0
*trnL(CUN)-Leu*	N	12492–12555	64	–	–	TAG	0
*rrnL*	N	12556–13855	1300	–	–	–	0
*trnV*	N	13856–13925	70	–	–	TAC	0
*rrnS*	N	13926–14676	751	–	–	–	0
*Control region*	–	14677–18682	4006	–	–	–	0

### Results and discussion

**Figure 1.**
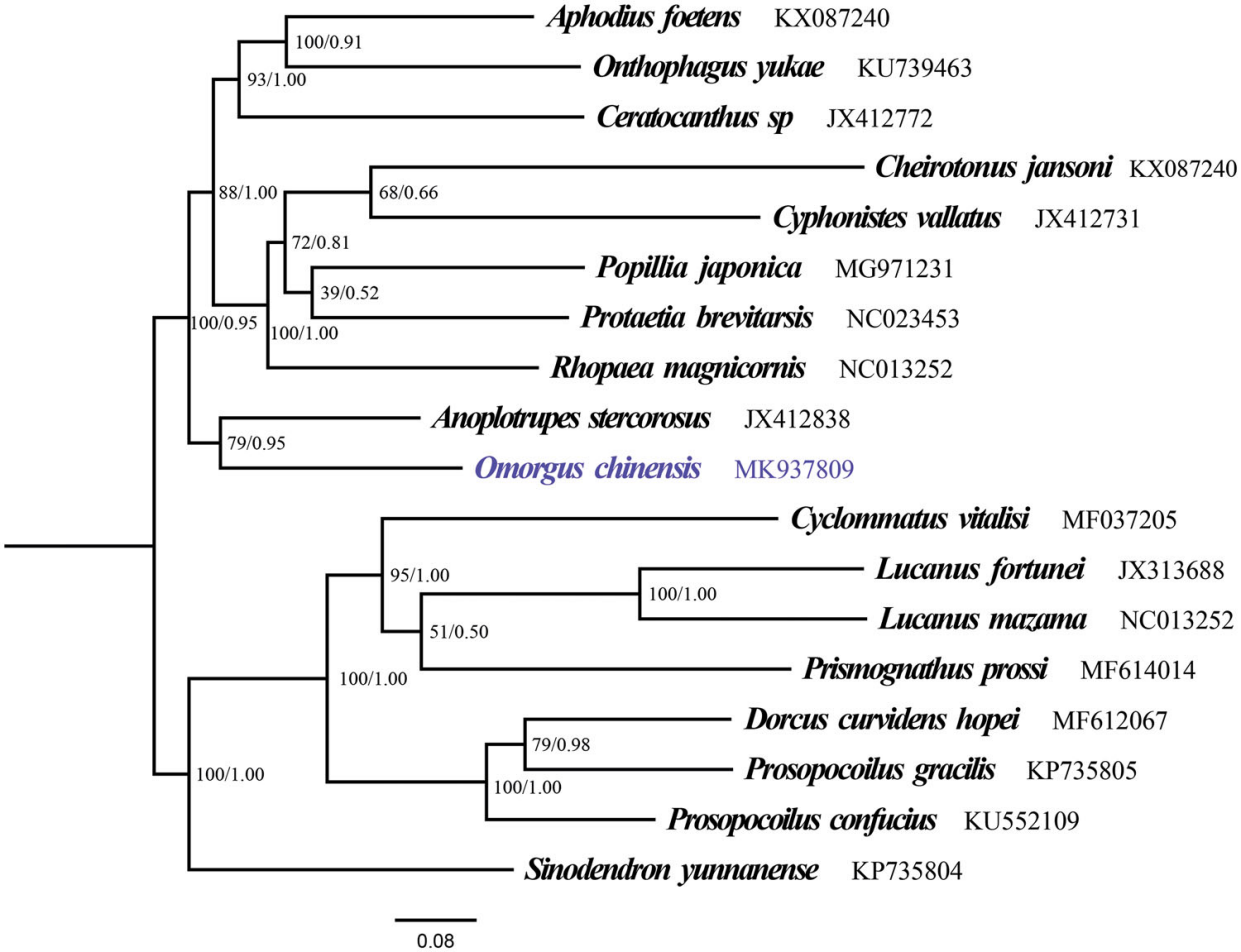
The ML and BI phylogenetic trees of *Omorgus chinensis* and 17 other scarabs based on 13 PCGs.

The mitogenome of *O. chinensis* is 18,682 bp in length, with the typical gene content as other known Coleoptera mitogenomes: 22 transfer RNA genes (tRNAs), 2 ribosomal RNA genes (16S rRNA and 12S rRNA), 13 protein-coding genes (PCGs) and 1 non-coding AT-rich region (Sheffield et al. 2009; Kim and Farrell 2015; Wu et al. 2016; Yang et al. 2016; Du et al. 2017). The base compositions of the mitogenome nucleotide composition were A (39.44%), C (13.82%), T (36.78%), and G (9.96%) with an AT content of 76.22%. AT-skew and GC-skews were calculated as 0.035 and −0.162. AT-biased were found in the protein-coding region (75.66%), rRNA (80.09%), tRNAs (78.11%) and control region (75.16%). As a typical invertebrate mitogenome, 12 PCGs of the sequence employ same initiation codons ATN, except the start codon of *COI* is AAT. 10/13 PCGs shared the typical termination codons TAA and TAG, while others use TA residue or a single T as the terminator codons. All of the 22 tRNAs range from 63 to 70 bp in length, 14 of the 22 tRNA-coding genes were located on the J-strand and others were located on the N-strand (Table 3). Secondary structures predicted by the tRNA scan-SE suggested that all the tRNA genes in *O. chinensis* adopted a typical clover-leaf structurewith the exception of tRNA-Ser (AGN), tRNA-Ser (AGN) has the deficiency of the dihydrouridine arm. which is a typical feature of metazoan mitochondrial genomes (Cameron 2014). The control region is 4006 bp long and is located between 12S rRNA and tRNA-Ile. ML and BI analyses obtained the consistent topology. The resolution of maximum likelihood and Bayesian analyses (Figure 1) both indicates that the newly sequenced species *O. chinensis* (Coleoptera: Trogidae) shared a close ancestor with *Anoplotrupes stercorosus* (Coleoptera: Geotrupidae) and closely related to Lucanidae.

In conclusion, the first complete mitogenome of Trogidae, *O. chinensis* is a typical metazoan mitogenome. Phylogenetic analysis show that Trogidae shares a close ancestry with Lucanidae and Geotrupidae.

## Data Availability

The genome sequence data that support the findings of this study are openly available in GenBank of NCBI at [https://www.ncbi.nlm.nih.gov] (https://www.ncbi.nlm.nih.gov/) under the accession MK937809. The associated BioProject, Bio-Sample numbers and SAR number are PRJNA759651, SAMN21181119 and SRR 16510902, respectively.
